# A Prospective Survey of Skin Manifestations in Children With Inborn Errors of Immunity From a National Registry Over 17 Years

**DOI:** 10.3389/fimmu.2021.751469

**Published:** 2021-09-30

**Authors:** Waleed Al-Herz, Mohammad Zainal, Arti Nanda

**Affiliations:** ^1^ Department of Pediatrics, Faculty of Medicine, Kuwait University, Kuwait City, Kuwait; ^2^ Allergy and Clinical Immunology Unit, Pediatric Department, Al-Sabah Hospital, Kuwait City, Kuwait; ^3^ Department of Quantitative Methods and Information Systems, College of Business Administration, Kuwait University, Kuwait City, Kuwait; ^4^ As’ad Al-Hamad Dermatology Center, Kuwait City, Kuwait

**Keywords:** skin manifestations, inborn errors of immunity, eczematoid rashes, cutaneous infections, autoimmunity, non-infectious cutaneous granulomas, immunodeficiency

## Abstract

**Background and Objectives:**

Reports on skin manifestations in inborn errors of immunity (IEI) are based on retrospective analysis, small series, or isolated case reports. The present prospective study aimed to determine the spectrum of skin manifestations in children with IEI and their relevance to specific molecular defects.

**Materials and Methods:**

The data were obtained from the Kuwait National Primary Immunodeficiency Disorders Registry during the period of 2004–2020.

**Results:**

A total of 313 pediatric cases of IEI, 71% diagnosed at molecular level, were registered with a cumulative follow-up period of 29,734 months. Skin manifestations were seen in 40.3% of the patients, and they were among the presenting manifestations in 33%. Patients with skin manifestations were older at both onset and diagnosis ages of IEI symptoms, but this was statistically significant for the latter only. The diagnosis delay was significantly longer in patients with skin manifestations. There was a statistically significant association between having skin manifestations and IEI category, being more common in patients with complement deficiencies, combined immunodeficiencies, and diseases of immune dysregulation. There was no statistically significant association between having skin manifestations and both gender and survival. Skin infections were the most frequent manifestations followed by eczema and autoimmune associations. Among IEI with more than 10 cases, skin lesions were a consistent finding in dedicator of cytokinesis 8 (DOCK8) deficiency, hyper IgE syndrome, ataxia-telangiectasia, and recombination activation gene (RAG)1 deficiency.

**Conclusions:**

Skin manifestations are common in IEI patients, and they had significant diagnosis delay and referral to specialists. Improvement of awareness about IEI is needed among pediatricians and dermatologists.

## Introduction

Around 6 million people worldwide are suggested to be suffering from inborn errors of immunity (IEI) (1). According to a recent review, a total of 104,614 have been reported from different registries, with molecular defects identified only in 13.2% (2). Hence, a great majority of IEI patients remain undiagnosed.

Skin manifestations have been reported in 40%–70% of the patients with IEI, and they are among the presenting features in majority of them (3–8). There are only a few studies that have focused on the whole spectrum of cutaneous manifestations seen among IEI patients (4–8). Most data are restricted to cutaneous manifestations of one disorder, case series, or isolated case reports (9). We had earlier published a prospective report on skin manifestations of primary immunodeficient children registered in the Kuwait National Primary Immunodeficiency Registry (KNPIDR) over a span of 6 years (6, 10). Now, 10 years later, we have 313 children diagnosed with IEI. With improved diagnosis facilities and genetic testing, molecular diagnosis has been settled in most of them.

The present study aimed to determine the frequencies and characteristics of skin manifestations in children with IEI and to determine their relevance to specific molecular defects. This will hopefully highlight the importance of such manifestations to help in early diagnosis and timely management of IEI patients.

## Materials and Methods

### Patients’ Data

The data were obtained from the KNPIDR as part of a study approved by The Research and Ethics Committee of the Ministry of Health in Kuwait and by the Kuwait University Health Sciences Center Ethical Committee in accordance with the *Declaration of Helsinki* (10). An informed written consent was obtained from patients and/or families for whom testing was done for research purposes. A written informed consent was obtained from the patients and/or families for publishing the images. The subjects were diagnosed between January 2004 and December 2020 and followed prospectively. The patients were followed prospectively by WA-H and AN whenever they showed skin manifestations. Patients were categorized according to the International Union of Immunological Societies (IUIS) Primary Immunodeficiency Diseases Committee Report on Inborn Errors of Immunity (2019) (11). Secondary immunodeficiencies were ruled out by obtaining a complete history and by performing proper testing when needed. The diagnosis of skin manifestations was made clinically and was supported by a skin biopsy and other relevant investigations whenever indicated.

### Statistics

Minitab 19.2020.1 (Minitab LLC, PA, USA) software was used to carry out all statistical tests. Pearson’s chi-square test was used to assess whether skin manifestations have any relation with death count, gender, or IEI categories. A two-sample t-test was used to assess whether there is a difference in onset age, diagnosis age, and diagnosis delay between the group of patients with and without skin manifestations. The Kaplan–Meier survival analysis was used to estimate the survival function. Survivals were calculated from the date of diagnosis until the date of death (uncensored) or until the end of the study period (censored). The p-value ≤0.05 was used as the cutoff level for statistical significance.

## Results

### Patients

A total of 313 children with IEI (162 males and 151 females) were diagnosed during the study period and followed prospectively. Two hundred twenty-two patients (71%) were diagnosed at the molecular level. The cumulative follow-up period for all patients was 29,734 months (2,477 years) [mean 95 months, standard deviation (SD) 65.59, 0–204 months]. The distribution of these patients according to IEI categories (11) showed predominance of immunodeficiencies affecting cellular and humoral immunity (CIDs) (33.54%), followed by combined immunodeficiencies with associated syndromic features (Sy-CIDs) (23%), diseases of immune dysregulation (15.01%), predominant antibody deficiencies (PADs) (12.46%), congenital defects of phagocyte number and functions (9.58%), complement deficiencies (5.11%), defects of intrinsic and innate immunity (0.63%), and autoinflammatory disorders and bone marrow failure (0.31% each). **Table 1** shows the frequencies of skin manifestations among the registered patients based on IEI categories.

**Table 1:** Frequency of skin manifestations in children with IEI registered in the Kuwait National Primary Immunodeficiency Registry.

Category	Total number and percentage (%) of patients	Number and percentage (%) of patients with skin manifestations
Immunodeficiencies affecting cellular and humoral immunity	105 (33.54)	52 (49.52)
Combined immunodeficiencies with associated or syndromic features	72 (23)	33 (45.83)
Predominantly antibody deficiencies	39 (12.46)	5 (12.82)
Diseases of immune dysregulation	47 (15.01)	18 (38.29)
Congenital defects of phagocyte number or function	30 (9.58)	8 (26.66)
Defects in intrinsic and innate immunity	2 (0.63)	0
Autoinflammatory disorders	1 (0.31)	1 (100)
Complement deficiency	16 (5.11)	9 (56.25)
Bone marrow failure	1 (0.31)	0
Total	313 (100)	126 (40.26)

IEI, inborn errors of immunity.

### Characteristics of Skin Manifestations

One hundred twenty-six (40.3%) patients were observed to have skin manifestations, and in 103 (33% of all patients and 82% of those with skin lesions), they were among the presenting signs. Patients with skin manifestations were older at both onset and diagnosis ages of IEI symptoms compared to those with no such manifestations, but this was statistically significant for the latter only (**Table 2**). The diagnosis delay was longer in patients with skin manifestations, and it reached the level of significance (**Table 2**). There was no statistically significant association between having skin manifestations and gender (66 males and 60 females, p = 0.856). **Table 3** shows the distribution of skin manifestations among the registered patients. Skin manifestations affected >50% of patients with complement deficiencies and almost half of patients with CID and Sy-CID. There was a statistically significant association between having skin manifestations and IEI category after merging together patients who belong to defects in intrinsic and innate immunity, autoinflammatory disorders, complement deficiencies, and bone marrow failure categories due to low numbers (p = 0.001) (**Table 1**).

**Table 2:** Onset age, diagnosis age, and diagnosis delay of patients with IEI with respect to having skin manifestations.

	Skin Manifestations	Total Count	Mean	SD	Median
Onset age^a^,^b^	–	187	13.95	35.90	3.00
	+	126	16.01	25.42	5.00
Diagnosis age^a^,^c^	–	187	32.40	58.24	8.00
	+	126	51.87	69.27	24.00
Diagnosis delay^a^,^d^	–	187	18.44	37.43	5.00
	+	126	35.86	59.50	10.00

a Months.

b p = 0.554.

c p = 0.01.

d p = 0.004.

IEI, inborn errors of immunity.

**Table 3:** Characterization of skin manifestations according to specific diagnosis among children with IEI registered in the KNPIDR.

IEI		Total	With Skin Manif-estations	Skin as a primary manifest-tation	Skin Manifestations							
					Infections	Eczema	Erythrode-rma	Alopecia	Granulom-as	**Specific to an IEI	***Autoimm-une	Miscellaneous
		N	N (%)	N (%)^+^	N (%)^#^	N (%)^#^	N (%)^#^	N (%)^#^	N (%)^#^	N (%)^#^	N (%)^#^	N (%)^#^
**All Groups**		**313**	**126 (40.3)**	**103 (82)**	**51 (40.5)**	**49 (39)**	**14 (11)**	**12 (9.5)**	**5 (4)**	**27 (21)**	**30 (24)**	**24 (19)**
	**All patients**	105	52 (49.52)	41 (79)	24 (46)	29 (56)	12 (23)	8 (15)	3 (6)	1 (2)	13 (25)	9 (17)
	**DOCK8 Deficiency**	10	10 (100)	10 (100)	9 (90)	9 (90)						2 (Mucosal pigmentation) (20)
	**RAG1 Deficiency**	11	9 (82)	8 (89)	5 (55.6)	7 (78)	7 (78)	5 (45.5)			6 (OS) (67)	2 (Nail dystrophy -1; skin peeling – 1) (22)
	**RAG2 Deficiency**	6	5 (83)	2 (40)	4 (80)			1 (20)	1 (20)		1 (20)	
	**MHCII Deficiency**	20	5 (25)	2 (40)		4 (80)	1 (20)					
	**DCLRE1C Deficiency**	7	3 (43)	2 (67)	2 (67)	1 (33)	1 (33)				1 (OS)	
	**JAK3 Deficiency**	5	2 (40)	2 (100)		2 (100)						
	**CD3δ Deficiency**	3	2 (67)	2 (100)			2 (100)	1 (50)	1 (50)		2 (OS) (100)	
	**ADA Deficiency**	2	2 (100)	2 (100)								2 (Non-specific hyperpigmentat-ion (100)
	**DOCK2 Deficiency**	3	2 (67)			1 (50)					1 (50)	
	**TFRC Deficiency**	8	2 (25)	2 (100)		2 (100)						–
	**AK2 Deficiency**	3	2 (67)	2 (100)		1 (50)						1 (Purpura fulminans) (100)
	**ZAP70 Deficiency**	2	1 (50)	1 (100)		1 (100)						–
	**ICOS Deficiency**	3	1 (33)	1 (100)		–				1		1 (ichthyosis) (100)
	**C-REL Deficiency**	1	1 (100)	1 (100)								1 (Nail dystrophy) (100)
	**CID (Unspecified)**	19	3 (16)	2 (67)	3 (100)	1 (33)			1 (33)			
	**OS (unspecified)**	2	2 (100)	2 (100)	1 (50)	–	1 (50)	1 (50)			2 (100)	
**Sy-CID**	All patients	72	33 (45.8)	28 (85)	17 (52)	17 (52)	2 (6)	1 (3)	0	12 (36)	2 (6)	4 (12)
	**Ataxia- Telangiectasia**	12	12 (100)	10 (83)	3 (25)	1 (8)				12 (100)		2 (Acanthosis nigricans -1; hypertrichosis – 1) (17)
	**STAT3 Deficiency**	4	4 (100)	4 (100)	4 (100)	4 (100)						
	**Hyper IgE Syndrome (unspecified)**	6	6 (100)	6 (100)	6 (100)	6 (100)	1 (17)					
	**Wiskott-Aldrich Syndrome**	3	3 (100)	3 (100)	1 (33)	3 (100)					1 (33)	1 (Erythema annulare centrifugum) (33)
	**STAT5B Deficiency**	2	2 (100)	2 (100)		2 (100)						
	**MYSM1 Deficiency**	5	2 (40)	1 (50)	1 (50)						1 (50)	
	**ICF**	4	1 (25)									1 (Dyspigmentation) (100)
	**RMRP deficiency**	1	1 (100)	1 (100)	1 (100)							
	**Comel- Netherton Syndrome**	1	1 (100)	1 (100)	–	1 (100)	1 (100)	1 (100)				
	**HOIP Deficiency**	1	1 (100)		1 (100)							
	**Others**	33	0									
**PAD**	**All patients**	39	5 (12.8)	5 (100)	3 (60)	2 (40)		2 (40)	1 (20)		3 (60)	3 (60)
	**NFKB2 Deficiency**	2	2 (100)	2 (100)	2 (100)	1 (50)		2 (100)			2 (100)	2 (Nail dystrophy – 2; AGEP – 1) (100)
	**CVID (Unspecified)**	9	1 (11)	1 (100)	1 (100)				1 (100)			
	**AID Deficiency**	9	2 (22)	2 (100)		1 (50)					1 (50)	
	**Others**	19	0									
**Diseases of immune dysregulation**	**All patients**	47	18 (38.3)	14 (78)	4 (22)	1 (5.6)		1 (5.6)		8 (44)	6 (33)	6 (33)
	**Primary Familial Hemophagocy-tic Lymphohistioc-ytosis**	21	6 (29)	6 (100)	1 (17)					2 (33)		3 (Non-specific skin rash – 3) (50)
	**Chediak-Hihgashi Syndrome**	4	4 (100)	4 (100)	1 (25)					4 (100)		1 (Facial erythema – 1) (25)
	Griscelli Syndrome	2	2 (100)	2 (100)						2 (100)		
	**LRBA Deficiency**	3	1 (33)								1 (100)	
	**APS-1 (APECED)**	3	1 (33)		1 (100)			1 (100)			1 (100)	1 (Nail dystrophy - 1) (100)
	**ALPS-FAS deficiency**	2	1 (50)								1 (100)	
	**Immune Dysregulation (Unspecified)**	4	3 (75)	2 (67)	1 (33)	1					3 (100)	1 (Skin peeling – 1) (33)
	**Others**	8	0									
**Congenital defects of phagocyte number or function**	**All patients**	30	8 (26.7)	7 (87.5)	3 (37.5)					5 (62.5)	1 (3.3)	1 (12.5)
	**Papillon Lefevre Syndrome**	5	5 (100)	5 (100)						5		
	**Glycogen Storage Disease Type 1b**	3	1 (33)		1 (6)							
	**Leukocyte Adhesion Defect**	1	1 (100)	1 (100)	1 (100)							1 (Delayed wound healing + scar formation – 1) (100)
	**CGD (p22phox)**	4	1 (25)	1 (100)	1 (100)						1 (100)	
	**Others**	17	0									
**Defects in intrinsic and innate immunity**	**INFg deficiency**	2	0									
**Autoinflammatory disorders**	**All patients**	1	1 (100)	1 (100)					1	1 (100)		1 (100)
	**Blau Syndrome**	1	1 (100)	1 (100)					1 (100)	1 (100)		1 (Angioma serpiginosum – 1) (100)
**Complement deficiency**	**All patients**	16	9 (56.25)	7 (78)							5 (56)	
	**C4 Deficiency**	5	5 (100)	3 (60)							5 (100)	
	**C1 Esterase Inhibitor Deficiency**	5	4 (80)	4 (100)						4 (100)		
	**Others**	6	0									
**Bone marrow failure**	**RTEL1 deficiency**	1	0									

^+#^ % of patients with skin manifestations.

** **Table 4**.

*** **Table 5**.

IEI, inborn errors of immunity; KNPIDR, Kuwait National Primary Immunodeficiency Registry; CID, immunodeficiencies affecting cellular and humoral immunity; Sy-CID, combined immunodeficiencies with associated syndromic features; DOCK8, dedicator of cytokinesis 8; DOCK2, dedicator of cytokinesis 2; STAT3, signal transducer and activator of transcription 3; RAG, recombination activation gene; MHCII, major histocompatibility complex II; JAK3, Janus kinase 3; CD3δ, cluster designation 3δ; ADA, adenosine deaminase; TFRC, transferrin receptor; AK2, adenylate kinase 2; ZAP70, zeta-chain-associated protein kinase 70; STAT5B, signal transducer and activator of transcription 5B; MYSM1, Myb-like, SWIRM and MPN domains 1; ICF, immunodeficiency, centromeric region instability, facial anomalies syndrome; RMRP, RNAse mitochondrial RNA processing; HOIP, HOIL-1 interacting protein; CVID, common variable immunodeficiency; AID, activation-induced cytidine deaminase; LRBA, LPS-responsive beige-like anchor; ALPS, autoimmune lymphoproliferative syndrome; CGD, chronic granulomatous disease; INFg, interferon gamma; RTEL1, regulator of telomere elongation helicase 1.

Among 126 patients with skin manifestation, 60 (47.6%) had one skin manifestation, 28 (22.2%) had two, and 38 (30.2%) had three or more during the course of the disease. Skin infections were the most prevalent, followed by eczema/eczematoid rashes and autoimmune skin associations (**Table 3**). Miscellaneous skin manifestations not peculiar to a specific IEI were seen in 19% of the patients with skin manifestations. Among specific IEI with more than 10 patients registered in the KNPIDR, skin manifestations were present in 100% of the patients with dedicator of cytokinesis 8 (*DOCK8)* deficiency, hyper IgE syndrome (HIGE) [both signal transducer and activator of transcription 3 (*STAT3)* deficiency and unspecified], and ataxia-telangiectasia (AT) and in 82% of recombination activation gene 1 (*RAG1*) deficiency, and they were among the presenting signs in 79%, 100%, 83%, and 89% of them, respectively (**Table 3**).

**Figure 1:**
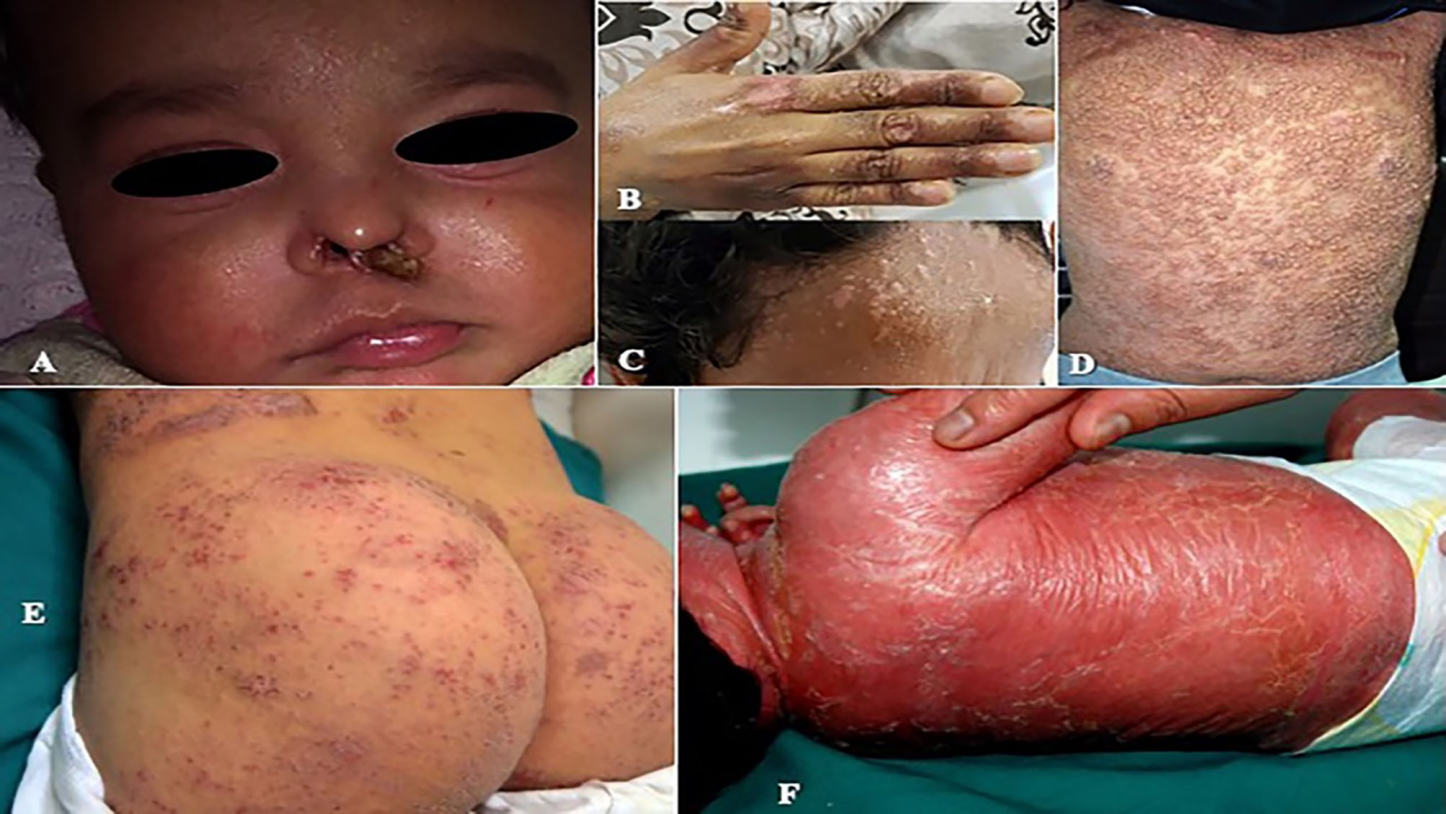
**(A)** Perinasal ecthyma gangrenosum in a patient with *DCLRE1C* deficiency. **(B, C)** Multiple recalcitrant warts in a patient with common variable immunodeficiency (CVID). **(D)** Generalized molluscum contagiosum in a patient with *dedicator of cytokinesis 8* (*DOCK8*) deficiency. **(E)** Severe eczematous rashes in a patient with *signal transducer and activator of transcription 3* (*STAT3*) deficiency. **(F)** Erythroderma in a patient with Omenn syndrome due to *recombination activation gene 1* (*RAG1*) deficiency.

Among infectious manifestations seen in 51 patients, bacterial infections were more prevalent and seen followed by viral and fungal infections (**Table 4**). Skin infections were prevalent among patients with Sy-CID followed by CID (**Tables 3**, **4**). Skin infections with widespread eczematous/eczematoid rashes were a consistent finding in all patients with HIGE. Abscess formation was seen in all patients with *STAT3* deficiency and DNA Cross-Link Repair 1C (*DCLREIC*) deficiency and in the majority of patients with *DOCK8* deficiency. Four patients (8%) were confirmed to have ecthyma gangrenosum (EG) due to *Pseudomonas* infection that was caused by either primary skin invasion or as part of secondary invasion following *Pseudomonas* septicemia (**Figure 1A**). Various viral infections including warts (**Figures 1B, C** **)** and molluscum contagiosum (MC) (**Figure 1D**) were observed to be more widespread and recurrent.

**Table 4:** Distribution of skin infections among 313 IEI children registered in the KNPIDR.

IEI Category		Total N of Patients	With skin manifestations	*With skin infections	Infections		
				N ^+^(%)	Bacterial	Viral	Fungal
All Groups		313	126	51 (16.3)	33 ^+^(10.6%)	19 ^+^(6%)	8 ^+^(2.6%)
**CID**	**Total**	105	52	24 (22.9)	19	8	1
	**DOCK8 deficiency**	10	9	9 (90)	9 (Impetigo/folliculitis 9, abscess formation 6, cellulitis 1)	3 (MC 2, warts 1, recurrent HSV infection 1)	
	**RAG1 deficiency**	11	9	5 (45.5)	5 (Impetigo/folliculitis 5)		
	**RAG2 deficiency**	6	5	4 (66.7)	2 (Impetigo 1, ecthyma gangrenosum 1)	2 (MC 2)	
	**DCLRE1C deficiency**	7	3	2 (28.6)	2 (Abscess formation 2, impetigo 1, ecthyma gangrenosum 1)		
	**CID unspecified**	19	3	3 (15.8)	1 (Ecthyma gangrenosum)	3 (Warts 2, disseminated HSV 1)	1 (Candidiasis)
	**Others**	53	22	0			
**Sy-CID**	**Total**	72	33	17 (23.6)	10	8	4
	**STAT3 deficiency**	4	4	4 (100)	4 (Impetigo/folliculitis 4, abscess formation 4, ecthyma gangrenosum 1)	1 (Severe chicken pox)	4 (Candidial paronychia and onychomycosis 4)
	**Hyper IgE syndrome (Unspecified)**	6	6	6 (100)	6 (Impetigo/folliculitis)		
	**Wiskott–Aldrich syndrome**	3	3	1 (33.3)		1 (MC 1)	
	**Ataxia-telangiectasia**	12	12	3 (25)		3 (MC 2, warts 2)	
	**MYSM1 deficiency**	5	2	1 (20)		1 (Warts)	
	**RMRP deficiency**	1	1	1 (100)		1 (Warts)	
	**HOIP deficiency**	1	1	1 (100)		1 (Warts)	
	**Others**	40	4	0			
	**Total**	39	5	3 (7.7)		2	1
	**NFKB2 deficiency**	2	2	2 (100)		1 (Warts + recurrent herpes zoster)	1 (Chronic mucocutaneous candidiasis)
	**CVID (unspecified)**	9	1	1 (11.1)		1 (Warts)	
	**Others**	28	2	0			
**Diseases of immune dysregulation**	**Total**	47	18	4 (8.5)	1	1	2
	**Primary familial hemophagocytic lymphohistiocytosis**	21	6	1 (4.8)			1 (Candidial onychomycosis)
	**Chediak-Higashi syndrome**	4	4	1 (25)	1 (Acute paronychia)		
	**APECED (APS -1)**	3	1	1 (33.3)			1 (Candidial onychomycosis)
	**Immune dysregulation (unspecified)**	4	3	1 (25)		1 (Warts)	
	**Others**	18	5	0			
**Congenital defects of phagocyte number or function**	Total	30	8	3 (10)	3		
	**Glycogen storage disease type 1**	3	1	1 (2)	1 (Abscess)		
	**Leukocyte adhesion defect**	1	1	1	1 (impetigo + staphylococcal scalded skin syndrome)		
	**CGD (p22phox)**	4	1	1 (2)	1 (Abscess)		
	**Others**	22	5	0			

*Total number includes patients with more than one skin infection in the same patient.

^+^% calculated from total number of IEI patients in respective category.

IEI, inborn errors of immunity; KNPIDR, Kuwait National Primary Immunodeficiency Registry; CID, immunodeficiencies affecting cellular and humoral immunity; Sy-CID, combined immunodeficiencies with associated syndromic features; PAD, predominant antibody deficiencies; DOCK8, dedicator of cytokinesis 8; STAT3, signal transducer and activator of transcription 3; RAG, recombination activation gene; NFKB2, nuclear factor kappa B subunit 2; TFRC, transferrin receptor; AK2, adenylate kinase 2; ZAP70, zeta-chain-associated protein kinase 70; STAT5B, signal transducer and activator of transcription 5B; MYSM1, Myb-like, SWIRM and MPN domains 1; RMRP, RNAse mitochondrial RNA processing; HOIP, HOIL-1 interacting protein; CVID, common variable immunodeficiency; LRBA, LPS-responsive beige-like anchor; ALPS, autoimmune lymphoproliferative syndrome; CGD, chronic granulomatous disease; INFg, interferon gamma; RTEL1, regulator of telomere elongation helicase 1.

**Figure 2:**
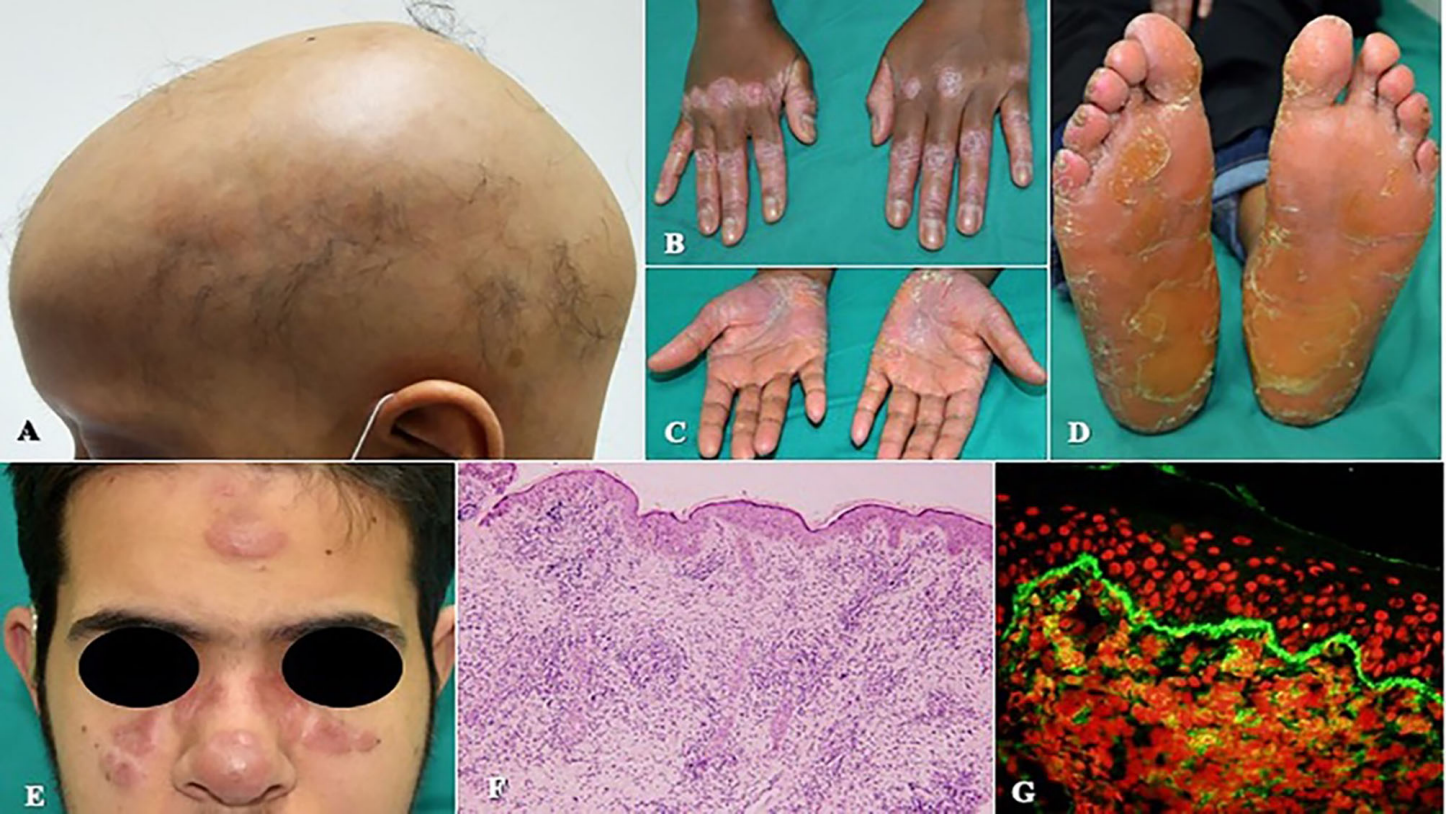
**(A)** Alopecia areata in a patient with *nuclear factor kappa B subunit 2* (*NFKB2*) deficiency. **(B–D)** Erythematous scaly plaques and palmoplantar keratoderma in a patient with Papillon–Lefèvre syndrome. **(E–G)** Tumid lupus erythematosus lesions on the face, skin histopathology showing features of lupus erythematosus, and direct immunofluorescence with granular deposition of IgM at the basement membrane zone in a patient with *activation-induced cytidine deaminase* (*AID*) deficiency.

Eczema and eczematoid rashes were more often seen in patients with CID and Sy-CID and were consistent signs in patients with HIGE and Wiskott–Aldrich syndrome (WAS) and *DOCK8* deficiency (**Figure 1E**). Among 14 cases with erythroderma, 12 (86%) were diagnosed with CID and was a consistent sign in patients with Omenn syndrome (OS) (**Figure 1F**). The other two cases with erythroderma included a case of HIGE and Comel–Netherton syndrome each. Among 12 cases of alopecia, eight were the patients with CID that included seven patients with OS. Three cases among other four were of alopecia areata (**Figure 2A**) associated with *NFKB* deficiency in two and autoimmune polyendocrinopathy candidiasis ectodermal dystrophy (APECED) syndrome in one. Among five patients with cutaneous granulomas, three were associated with CID and one each with common variable immunodeficiency (CVID) and Blau syndrome.

**Table 5** shows the specific skin manifestations seen in IEI patients. Mucocutaneous telangiectasia was observed to be a consistent feature in all patients with AT. Silvery gray hair and hypopigmentation of skin were consistent findings in patients with Chediak–Higashi syndrome and Griscelli syndrome; scaly rashes on the extremities, palmoplantar keratoderma (**Figures 2B**–**D**), tooth abnormalities, and gingivitis were consistent findings of Papillon–Lefevre syndrome.

**Table 5:** Specific skin manifestations among 27 children with IEI registered in the KNPIDR.

Diagnosis (N)	Specific Skin Manifestations
	N (%)
Ataxia-telangiectasia (12)	Telangiectasia
	12 (100)
Primary familial hemophagocytic lymphohistiocytosis (6)	Langerhans cell histiocytosis
	2 (33.3)
Chediak–Higashi syndrome (4)	Silvery gray hair + skin hypopigmentation
	4 (100)
Griscelli syndrome (2)	Silvery gray hair + skin dyspigmentation
	2 (100)
Papillon–Lefevre syndrome (5)	Palmoplantar keratoderma + psoriasiform skin rashes + tooth abnormalities
	5 (100)
Blau syndrome (1)	Sarcoidosis
	1 (100)
C1 esterase inhibitor deficiency (4)	Angioedema
	4 (100)

IEI, inborn errors of immunity; KNPIDR, Kuwait National Primary Immunodeficiency Registry.

Autoimmune skin diseases were seen among 9.5% of total IEI cases and were more often encountered in patients with complement deficiencies followed by diseases of immune dysregulation (**Table 6**, **Figures 2E–G**).

**Table 6:** Autoimmune skin associations with IEI among children registered in the KNPIDR.

IEI Category	Total N of Patients	With Skin Manifestations	With Autoimmune Skin Manifestations (N)
Immunodeficiencies affecting cellular and humoral immunity			
- RAG1 deficiency	11	9	Omenn syndrome (6)
- CD3δ deficiency	3	2	Omenn syndrome (2)
- DCLRE1C deficiency	7	3	Omenn syndrome (1)
- RAG2 deficiency	6	5	Psoriasis (1)
- DOCK2 deficiency	3	2	Vasculitis (1)
- CID (Unspecified)	19	3	Omenn syndrome (2)
Combined immunodeficiencies with associated or syndromic features			
- Wiskott–Aldrich syndrome	3	3	Vasculitis (1)
- MYSM1 deficiency	5	2	Neutrophilic panniculitis (1)
Predominantly antibody deficiencies			
- NFKB2 deficiency	2	2	Alopecia areata (2)
- AID deficiency (Hyper IgM Syndrome)	9	2	Lichen planus (1)
			Psoriasis (1)
			SLE (1)
Diseases of immune dysregulation			
- LRBA deficiency	3	1	Urticaria (1)
- APS-1 (APECED)	3	1	Alopecia areata (1)
- ALPS-FAS deficiency	2	1	Vasculitis (1)
- Immune dysregulation unspecified	4	3	Psoriasis (1)
			Vitiligo (1)
			DLE (1)
Congenital defects of phagocyte number or function			
- CGD (p22phox)	4	1	Livedoid vasculitis (1)
Complement deficiency			
- C4 deficiency	5	5	Urticaria (5)
			SLE (1)

IEI, inborn errors of immunity; KNPIDR, Kuwait National Primary Immunodeficiency Registry; CID, immunodeficiencies affecting cellular and humoral immunity; DOCK2, dedicator of cytokinesis 2; RAG, recombination activation gene; APECED, autoimmune polyendocrinopathy candidiasis ectodermal dystrophy; SLE, systemic lupus erythematosus; DLE, discoid lupus erythematosus; NFKB2, nuclear factor kappa B subunit 2; CD3δ, cluster designation 3δ; MYSM1, Myb-like, SWIRM and MPN domains 1; AID, activation-induced cytidine deaminase; LRBA, LPS-responsive beige-like anchor; ALPS, autoimmune lymphoproliferative syndrome; CGD, chronic granulomatous disease.

### Survival Analysis

**Figure 3:**
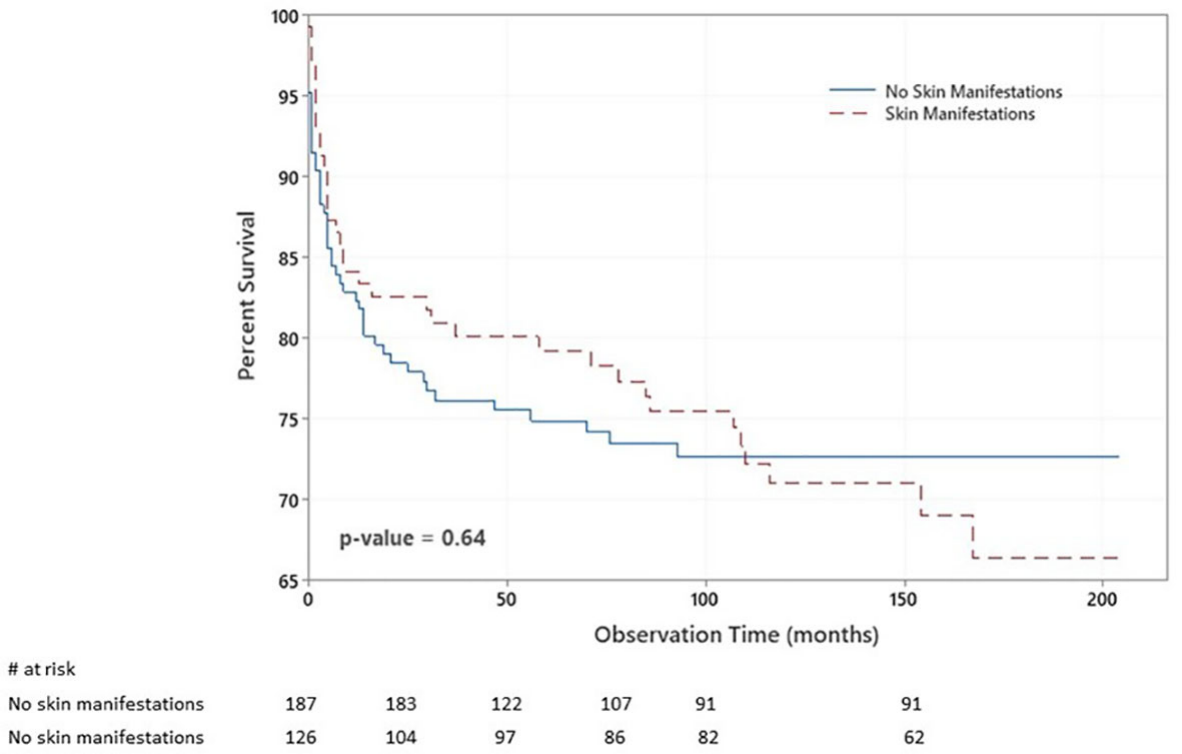
Kaplan–Meier survival plot of the chance of survival among children with inborn errors of immunity (IEI) with respect to having skin manifestations. The probabilities that patients with no skin manifestations survived 2, 6, and 14 years after diagnosis were 77%, 74%, and 72%. At the same time, they were 82%, 74%, and 42% among patients with skin manifestations.

There were 85 deaths during the study period. There was no significant statistical association between having skin manifestations and death (p = 0.644). **Figure 3** shows the Kaplan–Meier survival curves of children with IEI with respect to having skin manifestations.

## Discussion

The present study aimed to determine the frequencies and characteristics of skin manifestations in children with IEI who were followed prospectively over 17 years and registered in a national registry and to determine their relevance to specific molecular defects. Skin is an important organ affected in IEI patients, and skin manifestations are the presenting features in a vast number of patients. As the list of IEI continues to widen, so is expected the spectrum of cutaneous manifestations.

Compared to our previous study (6), the number of patients in the current report is doubled and they are more characterized at the molecular level. Hence, the frequency and characteristics of skin manifestations in IEI are better defined. An important finding of our report is that the IEI patients with skin manifestation had longer delay in diagnosis compared to patients with no such manifestations. This is probably due to un-awareness of primary physicians, general pediatricians and dermatologist that such manifestations could be caused or related to IEI and the presumption that they could be due to common skin diseases of childhood like atopic dermatitis and/or skin infections. This signifies delay in clinical suspicion of IEI and referral to the specialists and warrants to develop more awareness among the primary physicians, general dermatologists and the pediatricians.

We observed skin manifestations in 40% of our patients, and they were the presenting signs in 33%, representing 82% of all with skin manifestations. This high number compared to previous reports (4, 5, 7, 8) is partly due to the prospective type of our study and due to the relatively high number of patients with combined immunodeficiencies registered in the KNPIDR. Skin manifestations were observed to be a consistent finding in diseases with more than 10 patients registered like *DOCK8* deficiency, HIGE, *RAG1* deficiency, and AT. However, no definite inference can be drawn in diseases with small number of patients.

The prevalence of skin infections in the present report (16.3%) is much lower than that reported from Colombia, Tunisia, Iran, and Mexico (56.63%, 36.55%, 47.1%, and 61.5%, respectively) (4, 5, 7, 8) as well as from that reported by us earlier from Kuwait (30%) (6). This represents improved awareness of infection control care of our IEI patients over the years. Skin infections were significantly more often seen in patients with Sy-CID and CID as compared to other groups of IEI. This can be explained by the more severe type of immune defects affecting patients in these categories. Similar to our earlier observations and those reported from elsewhere (4–9, 12), *Staphylococcus aureus* abscess formation was a consistent feature in HIGE syndrome and *DOCK8* deficiency patients. Besides the common staphylococcal skin infections, patients are also prone to get rare infections due to Gram-negative bacteria that may result in EG, which has been reported earlier in patients with X-linked agammaglobulinemia, chronic granulomatous disease, and hemophagocytic lymphohistiocytosis (13–15).

Eczematous rashes have been reported in 13%–57% of the patients with IEI in previous reports (4–8). We feel that the low frequency of eczematous/eczematoid rashes in this report (15.7%) as compared to our earlier report (19%) (6) is due to widening of our IEI registry including several patients with IEI that are not associated with eczematous rashes. However, comparable to our earlier observations and those reported from elsewhere (4–9, 12), eczema is a consistent feature of HIGE syndrome and WAS and a predominant feature in patients with CID including *DOCK8*, *RAG1*, and MHC II deficiency.

Non-infectious cutaneous granulomas are a rare manifestation of IEI. Although they have been reported with several diseases, they are more often seen in patients with AT, CID, and CVID (16, 17). They were reported to constitute 0.77% of total IEI children in a report from France (17). Five patients with cutaneous granulomas in the present report were observed to constitute 1.6% of all patients with IEI. Four of these patients have been earlier reported (18).

Manifestations specific to various IEI were seen in 21% of the patients. These are the manifestations that can be seen in patients other than IEI, but when present in patients who are suspected to have an IEI, they raise the suspicion of a specific disease that needs to be ruled out.

Autoimmune manifestations related to skin, hair, and nail were second common after cytopenias were previously reported and constituted 21% of the patients with all autoimmune manifestations (19). Autoimmune diseases have been reported to be more frequent by a factor of 10 in patients with IEI than the general population, they tend to occur throughout the lifetime, and they have a prognostic significance (20). We observed autoimmune skin manifestations among 9.5% of patients with IEI, and they represented 24% of total patients with skin manifestations. They were more often seen in patients with complement deficiencies, followed by diseases of immune dysregulation, CID and PAD. The high prevalence of autoimmune diseases in IEI demonstrates the intricate relationships between the mechanisms involved in these two conditions and is a result of defects in central and peripheral tolerance with the influence of chronic and recurrent infections (21).

To conclude, skin is a common organ affected in the IEI, and being apparent can be more important than any other organ in terms of providing a first clue to diagnosis and thus in aiding in early detection and management of these patients. The strength of the current study is the inclusion of a relatively large number of molecularly defined patients who were followed prospectively over a long period of time by the same investigators. Limitations of the present study include the small number of cases of various individual IEI among most groups and restriction to one ethnic group that may not be representative of the true spectrum of IEI and their skin manifestations across the globe. Hence, more studies, both global and from different regions that include patients with different IEI or disease specific, are needed to have a better understanding of the effects of IEI on the skin.

## Data Availability Statement

The raw data supporting the conclusions of this article will be made available by the authors without undue reservation.

## Ethics Statement

The studies involving human participants were reviewed and approved by The Research and Ethics Committee of the Ministry of Health in Kuwait, and by the Kuwait University Health Sciences Center Ethical Committee, in accordance with the Declaration of Helsinki. An informed written consent was obtained from patients and/or families for whom testing was done for research purposes. Written informed consent for participation was not required for this study in accordance with the national legislation and the institutional requirements. Written informed consent was obtained from the individuals, and minors’ legal guardian/next of kin, for the publication of any potentially identifiable images or data included in this article.

## Author Contributions

WA-H contributed to the establishment of the KNPIDR, patients’ diagnosis, data collection and analysis, writing the article, approval of the submitted article, and agreement to be accountable for the content of the work. MZ contributed to the data analysis and statistics, approval of the submitted article, and agreement to be accountable for the content of the work. AN participated in patients’ diagnosis, data collection and analysis, writing the article, approval of the submitted article, and agreement to be accountable for the content of the work. All authors contributed to the article and approved the submitted version.

## Conflict of Interest

The authors declare that the research was conducted in the absence of any commercial or financial relationships that could be construed as a potential conflict of interest.

## Publisher’s Note

All claims expressed in this article are solely those of the authors and do not necessarily represent those of their affiliated organizations, or those of the publisher, the editors and the reviewers. Any product that may be evaluated in this article, or claim that may be made by its manufacturer, is not guaranteed or endorsed by the publisher.

## Abbreviations

IEI, inborn errors of immunity; KNPIDR, Kuwait National Primary Immunodeficiency Registry; IUIS, International Union of Immunological Societies; SD, standard deviation; CID, immunodeficiencies affecting cellular and humoral immunity; Sy-CID, combined immunodeficiencies with associated syndromic features; PAD, predominant antibody deficiencies; DOCK8, dedicator of cytokinesis 8; DOCK2, dedicator of cytokinesis 2; STAT3, signal transducer and activator of transcription 3; RAG, recombination activation gene; EG, ecthyma gangrenosum; MC, molluscum contagiosum; WAS, Wiskott–Aldrich syndrome; OS, Omenn syndrome; HIGE, hyper IgE syndrome; APECED, autoimmune polyendocrinopathy candidiasis ectodermal dystrophy; AT, ataxia telangiectasia; SLE, systemic lupus erythematosus; DLE, discoid lupus erythematosus; NFKB2, nuclear factor kappa B subunit 2; MHCII, major histocompatibility complex II; JAK3, Janus kinase 3; CD3δ, cluster designation 3δ; ADA, adenosine deaminase; TFRC, transferrin receptor; AK2, adenylate kinase 2; ZAP70, zeta-chain-associated protein kinase 70; STAT5B, signal transducer and activator of transcription 5B; MYSM1, Myb-like, SWIRM and MPN domains 1; ICF, immunodeficiency, centromeric region instability, facial anomalies syndrome; RMRP, RNAse mitochondrial RNA processing; HOIP, HOIL-1 interacting protein; CVID, common variable immunodeficiency; AID, activation-induced cytidine deaminase; LRBA, LPS-responsive beige-like anchor; ALPS, autoimmune lymphoproliferative syndrome; CGD, chronic granulomatous disease; INFg, interferon gamma; RTEL1, regulator of telomere elongation helicase 1.
